# Disease course of Chronic Relapsing Inflammatory Optic Neuropathy (CRION) in a single care center

**DOI:** 10.1590/0004-282X-ANP-2021-0157

**Published:** 2022-02-21

**Authors:** Luis Enrique MOLINA-CARRIÓN, Josehp LIRA-TECPA, María Pilar JIMÉNEZ-ARELLANO, María Pilar CRUZ-DOMÍNGUEZ, Gabriela MEDINA

**Affiliations:** 1 Hospital de Especialidades Centro Médico Nacional “La Raza”, Instituto Mexicano Del Seguro Social, Neurology Department, Mexico City, Mexico.; 2 Universidad Popular Autónoma del Estado de Puebla, Puebla, Mexico.; 3 Universidad Veracruzana, Veracruz, Mexico.; 4 Hospital de Especialidades Centro Médico Nacional “La Raza”, Instituto Mexicano del Seguro Social, Research Division, Mexico City, Mexico.; 5 Universidad Nacional Autónoma de México, Mexico City, Mexico.; 6 Hospital de Especialidades Centro Médico Nacional “La Raza”, Instituto Mexicano del Seguro Social, Translational Research Unit, Mexico City, Mexico.

**Keywords:** Optic Neuritis, Recurrence, Therapeutics, Neuritis Óptica, Recurrencia, Terapéutica

## Abstract

**Background:**

Chronic relapsing inflammatory optic neuropathy (CRION) is a recurrent, idiopathic optic neuritis and is considered as a rare disease.

**Objective:**

To describe the clinical course during long-term follow-up of patients with a diagnosis of CRION.

**Methods:**

From a cohort of 1,735 patients with demyelinating disorders, we selected patients aged over 16 years with CRION according to current criteria. Demographic and clinical data, including initial presentation, symptoms, number of relapses, time delay in diagnosis, diagnostic methods, and treatment were obtained from clinical files. Infections, autoimmune diseases, and multiple sclerosis, among other conditions, were ruled out in all patients.

**Results:**

We analyzed 30 patients with CRION: 24 women and six men, with mean age of 42.8±10.2 years, median disease course of 7.9 years (5.29-13.1), and median number of attacks of 2 (IQR 2-4). The initial manifestation was ocular pain in 97% and bilateral and sequential affection in 87%. Visual acuity was recovered in 50%, did not improve in 33%, and recovered incompletely in 17%. Antibodies against aquaporin-4 (AQP4-Abs) were negative in 73.3%. Magnetic resonance imaging of the brain was normal in 76.7%. None of the patients evolved to another demyelinating disease over time. Initial treatment was methylprednisolone in 100%, and plasmapheresis in 20%. Currently, all patients are on maintenance treatment with mycophenolate mofetil or rituximab with a decrease in relapsing rate.

**Conclusions:**

Diagnosis of CRION is challenging and should be kept in mind. Prompt diagnosis, adequate treatment and close follow-up are essential to prevent disabling sequelae in these patients.

## RESUMEN

**Antecedentes:**

Neuropatía óptica inflamatoria crónica recidivante (CRION) es una neuritis óptica idiopática recurrente, considerada una enfermedad rara.

**Objetivo:**

Describir la evolución clínica durante el seguimiento a largo plazo de pacientes con diagnóstico de CRION.

**Métodos:**

De una cohorte de 1.735 pacientes con trastornos desmielinizantes, seleccionamos pacientes mayores de 16 años con diagnóstico de CRION según los criterios actuales. Datos demográficos y clínicos, incluyendo presentación inicial, síntomas, recaídas, tiempo de retraso diagnóstico, métodos de diagnóstico y tratamiento se obtuvieron de los archivos clínicos. Se descartaron en todos los pacientes infecciones, enfermedades autoinmunes, esclerosis múltiple, entre otras condiciones.

**Resultados:**

Se analizaron 30 pacientes con diagnóstico de CRION: 24 mujeres y 6 hombres, edad media de 42,8±10,2 años, mediana del curso de la enfermedad de 7,9 años (5,2-13,1), mediana del número de recaídas 2 (IQR 2-4). La manifestación inicial fue dolor ocular en el 97% y afección bilateral y secuencial en el 87%. La agudeza visual mejoró en el 50%, sin recuperación en el 33% y con restauración incompleta en el 17%. Los anticuerpos contra acuaporina-4 (AQP4-Abs) fueron negativos en el 73,3%. La resonancia magnética cerebral fue normal en el 76,7%. Ningún paciente evolucionó hacia otra enfermedad desmielinizante en el seguimiento. El tratamiento inicial fue metilprednisolona en el 100%, y plasmaféresis en el 20%. Actualmente, todos los pacientes están en tratamiento de mantenimiento con micofenolato de mofetilo o rituximab con disminución de la tasa de recaídas.

**Conclusiones:**

El diagnóstico de CRION representa un desafío y debe tenerse en cuenta. El diagnóstico oportuno, tratamiento adecuado y seguimiento estrecho son fundamentales para evitar secuelas invalidantes.

## INTRODUCTION

Chronic relapsing inflammatory optic neuropathy (CRION) is a type of recurrent optic neuropathy of idiopathic origin that usually responds to treatment with systemic steroids or immunosuppressants and presents relapses upon withdrawal or tapering dose^1^. It was first described by Kidd et al.^2^. This disease is found worldwide, affecting more women than men, but the etiology remains unknown. The clinical presentation is characterized by unilateral or bilateral optic neuropathy, severe and persistent pain followed by subacute visual loss^3^, with both optic nerves affected and a latency period between attacks of days to years^3^ ^,^ ^4^.

Its diagnosis requires excluding other causes of optic neuritis, such as multiple sclerosis, granulomatous optic neuropathy secondary to sarcoidosis, tuberculosis or infections, and systemic autoimmune diseases. A good clinical history, physical examination, and ancillary diagnostic tests are necessary to reach a correct diagnosis^3^.

Although there is no consensus on the diagnostic criteria of CRION, five diagnostic criteria have been proposed: 1. a history of optic neuropathy and at least one relapse; 2. objective clinical evidence of loss of visual function; 3. seronegative antibodies against aquaporin-4 (AQP4-Abs); 4. imaging contrast enhancement of the acutely inflamed optic nerves; 5. response to immunosuppressive treatment and relapse after discontinuation or tapering of immunosuppressors^3^. Long-term follow-up is of great importance to evaluate disease course and progression, and information on this topic is limited due to the rarity of the disease. Therefore, we aimed to describe the clinical course in CRION patients.

## METHODS

From a cohort of 1,735 patients with demyelinating diseases including multiple sclerosis, neuromyelitis optica spectrum disorder (NMOSD), Baló’s concentric sclerosis, Schilder’s disease, and other inflammatory demyelinating diseases followed in the demyelinating diseases clinic in the Neurology department, in a tertiary level hospital in Mexico City, we selected patients with the diagnosis of CRION according to the criteria proposed by Petzold et al.^3^, both sexes, and aged over 16 years. Exclusion criteria were clinical files with missing 90% of data or loss to follow-up, infectious diseases, sarcoidosis, toxic, nutritional or hereditary neuropathies, the appearance of autoimmune diseases such as systemic lupus erythematosus, other demyelinating diseases, paraneoplastic syndrome, among others, during follow-up.

The clinical and laboratory data were obtained from hospital files. Clinical data included initial presentation, symptoms, number of relapses, delay in diagnosis, diagnostic methods, and treatment. The local Ethics and Investigation Committee approved the protocol. This investigation followed the guidelines of the Declaration of Helsinki, and it was considered without risk because retrospective documentary research methods were used. Data collected in the course of this investigation were kept confidential.

Descriptive statistics included mean, standard deviation, medians and IQR (interquartile range), and percentages. The Kaplan-Meier analysis was used to evaluate relapses during follow-up and after immunosuppressant treatment and Kruskal Wallis test was used to analyze time between relapses. Data were analyzed in *Statistical Package for the Social Sciences* (SPSS) v. 23 for Windows (IBM Corp. Chicago IL).

## RESULTS

From January 2010 to May 2021, a total of 30 cases diagnosed with CRION were treated in the demyelinating diseases clinic, 24 women and 6 men, with a mean age of 42.8±10.2 years, age at diagnosis of 38.6±11.1 years, age at onset of 35±11.1 years, and a median disease course of 7.95 years (IQR 5.29-13.1). Comorbidities were present in 7 patients (23%): 2 patients had hypothyroidism and the others had arterial hypertension, type 2 diabetes mellitus, choriocarcinoma, myasthenia gravis, glaucoma, and anxiety disorder.

Twenty-nine of 30 patients (97%) had ocular pain as the first clinical symptom, 26 (87%) had bilateral and sequential involvement, and 4 patients had only one eye affected. At the onset, 11 patients had left optic neuropathy, 13 had right optic neuropathy, and 6 bilateral optic neuropathy, with a latency period of 38 months (days to 34 years) between attacks in both eyes. Some patients were not diagnosed previously, with a median delay in diagnosis of 2 years (range 0-4 years). All patients had visual loss; 23 (77%) had a very severe visual loss (from 20/100 to 20/800). The median number of attacks was 2 (IQR 2-4); 15 (50%) had two, 7 (23.3%) had three, and 8 (26.6 %) had four or more, including the first episode. We observed a seasonal prevalence of relapses in 21 patients (70%) in fall and winter.

**Figure 1.**
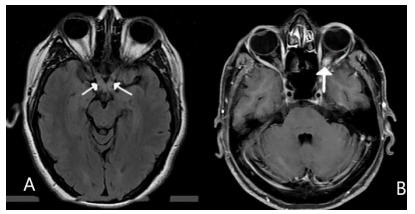
(A) Magnetic resonance imaging in Fluid-Attenuated Inversion Recovery sequence with hyperintensity in both optic nerves in the chiasmatic region. (B) Sequence in T1 with gadolinium with reinforcement of intraocular part of left optic nerve.

Fourteen patients (47%) had positive antinuclear antibodies, 13 patients had titers of 1:80, and one patient had titer of 1:160. These patients did not have clinical data of other autoimmune diseases, such as systemic lupus erythematosus, Sjögren syndrome, rheumatoid arthritis, or Behçet disease. Furthermore, other diagnoses such as multiple sclerosis, Lyme disease, tuberculosis, sarcoidosis, toxic, nutritional, hereditary, ischemic or compressive optic neuropathies were ruled out in all cases during follow-up. Seventy-three percent of patients were negative for AQP-4 antibodies, one patient was positive for AQP-4 antibodies, and the others were undetermined (Table 1). Most patients had a normal brain and spine MRI, and 16.7% were compatible with optic neuritis (hyperdensity of one or both optical nerves and enhancement with gadolinium) (Figures 1A and 1B). Twenty-three patients (76.7%) had alterations in visual evoked potentials, with axonal damage and demyelinating pattern (Table 1).

**Table 1.** Complementary studies.

Complementary studies			
Anti-AQP4 antibodies		Negative	22 (73.3%)
		Positive	1 (3.3%)
		Undetermined	7 (23.3%)
**ANA**			
1:80	13 (41.9%)	Fine speckled	12 (38.7%)
1:160	1 (3.2%)	Coarse speckled	1 (3.2%)
		Homogeneous	1(3.2%)
Brain and spine MRI		Normal	23 (76.7%)
		Optic neuritis	5 (16.7%)
		Microangiopathy	2 (6.7%)
Visual evoked potentials		Axonal	18 (60%)
		Demyelinating	12 (40%)

ANA: antinuclear antibodies; MRI: magnetic resonance imaging.

Initially, all patients received methylprednisolone pulses, and due to lack of response, 6 patients received plasmapheresis. Subsequently, they received different treatments such as azathioprine, rituximab, or mycophenolate mofetil. Initial and maintenance treatments are described in Table 2. Currently, all patients receive immunosuppressive treatment with mycophenolate mofetil or rituximab, and a decrease in the number of relapses has been observed, although there have been relapses with dose changes or withdrawal when patients have been referred to another center. After treatment, half of the patients had visual recovery, and the others had incomplete or no visual recovery (Table 2).

**Table 2.** Treatment, visual restoration and Expanded Disability Status Scale.

Treatment	Initial	Methylprednisolone	30 (100%)
		Plasmapheresis	6 (20%)
	Maintenance	Prednisone	22 (73.3%)
		Azathioprine	4 (13.3%)
		Rituximab	3 (10%)
		Interferon	1 (3.3%)
		Glatiramer acetate	1 (3.3%)
	Current treatment	Mycophenolate mofetil	26(86.6%)
		Rituximab	4 (13.3%)
Visual restoration	Restoration		15 (50%)
	No recovery		10 (33%)
	Incomplete restoration		5 (17%)
EDSS scale	Basal		3.0
	After 6 months		3 (2-3)
	After 1 year		3 (1-3)
	After 2 years		2 (0-3)
	Current		2 (0-3)

EDSS: Expanded Disability Status Scale. Data are expressed in medians and range.

**Figure 2.**
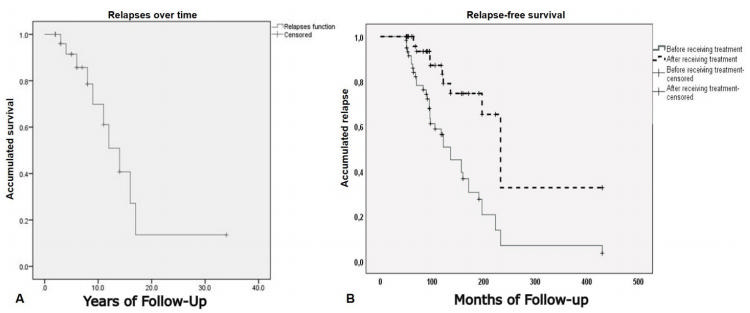
Kaplan Meier analysis of relapses.

The Kaplan-Meier estimate plot for relapses over time is shown in Figure 2A. Of note, most of the patients relapsed in the first 10 years. The annualized relapse rate was calculated as the total number of relapses divided by the total follow-up time per patient. There were 45 relapses before treatment and 13 relapses after treatment in the 30 patients, so the annualized relapse rate before treatment started was 4.5 relapses per year compared with 1.4 relapses per year after treatment with mycophenolate mofetil or rituximab (p=0.0001) (Figure 2B). The Kruskal Wallis test was used to analyze the time between relapses during follow-up. The time-interval varied from 2.5 to 23.5 months (p<0.001). In patients with more than 4 relapses, the interval time decreased with increasing number of relapses. There was a significant difference in the time between in the 1st and 2nd and in the 4^th^ and 5th relapses (p<0.01) (Table 3).

**Table 3.** Median time between relapses

Relapses	Median (IQR, months)
0 (Basal)	0
1	12.5 (2-37.7)
2	23.5* (3.2-42.7)
3	13.0 (3.2-77.2)
4	18.0
5	2.5** (0-7)
6	4.5 (2-7)

IQR: interquartile range; *between 1^st^ and 2^nd^ relapse (p=0.01); **between 4^th^ and 5^th^ relapse (p=0.04).

## DISCUSSION

In this retrospective cohort, we described the clinical course of patients with CRION. We found that most patients had bilateral and sequential optic neuritis with decreased latency between attacks in both eyes and a decrease in the number of relapses when mycophenolate mofetil or rituximab was used. We found a shorter time between attacks in both eyes in most of our patients, and the majority relapsed in the first 10 years of disease, findings similar to Kidd et al.^2^, that reported that most cases of optical affection were present in both eyes in a sequential way, ranging from days to 14 years. Also, we found a female predominance over males, as reported by Petzold et al.^3^.

Regarding visual acuity, we had a similar proportion of patients with an acuity lower than 20/100, in agreement with the findings of Kidd et al.^2^. CRION can be diagnosed with at least one relapse, and dependence on immunosuppressants is a cardinal finding^3^. Petzold et al.^3^ described 122 cases of CRION with varying results: 34 patients had less than 5 relapses, 11 had 5 to 10 episodes, and only one case had 18. In our study, most patients had at least 3 attacks, although we had one case with 7 relapses. We had one particular case of a patient with a very long latency period, with her first episode in 1984 and her second episode 34 years later.

There is no recent information about the seasonal prevalence of relapses. Koraszewska-Matuszewska et al.^5^, found a seasonal pattern in young patients with optic neuritis with a higher prevalence in fall and winter, similar to our patients. They also found a relationship with viral infections and trauma; however, these cases are related to optic neuritis and not CRION.

AQP4 antibodies can be present in patients with neuromyelitis optica and in a low percentage of classical multiple sclerosis^6^; in CRION, most patients are seronegative for AQP4-Abs, but a subset that can be positive^3^. In another study by Petzold et al.^7^, the presence of AQP4-Abs in different illnesses was compared [neuromyelitis optica, multiple sclerosis, CRION, relapsing isolated optic neuropathy (RION) and single isolated optic neuropathy (SION)]. They found seropositivity for these antibodies, which in CRION was 5%, similar to that reported by Jarius et al.^8^, with a worse visual outcome and a higher risk of conversion to neuromyelitis optica. We had only one patient positive for AQP4-Abs. Recently, the myelin oligodendrocyte glycoprotein antibodies (MOG-IgG) have been used for diagnosis and prognosis of CRION patients. Liu H et al.^9^ evaluated the status of MOG-IgG in 33 patients with CRION and found that 66.7% of them had MOG-IgG antibodies while 33.3% were seronegative for both AQP4-Abs and MOG-IgG. Patients positive for MOG-IgG had bilateral involvement more often and higher relapse rates than seronegative CRION patients. Petzold et al.^10^ reevaluated the cohort of patients with multiple sclerosis, CRION, RION, and SION and the prevalence of AQP4-Abs increased from 5% to 22% of patients. In comparison, MOG-IgG was identified in only 25% of patients with CRION after a long follow-up, and the prognosis prognosis of visual acuity was worse in patients seropositive for AQP4-Abs. In our series, the patient positive for AQP4-Abs had bilateral involvement, 3 relapses, very severe visual loss, and no visual recovery after treatment. Concerning anti-MOG antibodies, these tests are not available in our institution.

The cornerstone of CRION treatment is steroids, which responds well in most patients and can be used for a long time to maintain remission^3^. Nevertheless, frequent relapses require other immunosuppressants. Stiebel-Kalish et al.^11^ reported 6 cases of CRION treated initially with steroids and later with intravenous immunoglobulins who had a good response. Papp et al.^12^ described a case of CRION with anti-MOG antibodies who did not respond to steroids or other immunosuppressants but who responded well to infliximab. All our patients were treated with steroids in the acute phase and with immunosuppressants in the long-term management to avoid the adverse effects of corticosteroid therapy. There is insufficient information about mycophenolate mofetil and its effectiveness in CRION. Sahraian et al.^13^ recommended a dose of mycophenolate mofetil between 1000 and 3000 mg per day for management of NMOSD. Furthermore, they recommended azathioprine, mycophenolate mofetil, or rituximab as first-line treatment for NMOSD. There is limited information on the use of rituximab in patients with CRION; one patient with 4 relapses in 6 months despite several treatments responded to rituximab with a tapering dose of prednisolone without new relapses in 6 months^14^.

The differential diagnosis of CRION and RION is essential, as the latter is not steroid-dependent^15^. All our patients are currently on immunosuppressants, and they have relapsed with dose changes or withdrawal.

It is often difficult to distinguish simultaneous bilateral loss from sequential visual loss, as symptoms in one eye may be so severe that minimal symptoms in the other eye may be missed. Therefore, clinical data remains an essential factor in the diagnosis of this rare entity^2^ ^,^ ^16^.

Our study has some limitations; one of them is that not all patients had a determination of AQP4 antibodies and other antibodies such as anti-MOG. In this regard, a recent study found that a subset of CRION patients with MOG-IgG had more severe disease course^17^. Another limitation is that some patients may later develop other diseases; for example, Stiebel-Kalish et al.^11^ found 3 patients with other conditions, one with SLE and another with Wegener’s granulomatosis, which developed within one year of follow-up, and one had SLE five years later. We did not perform coherence optic tomography, as it is not available in our hospital. This study provides images with a resolution similar to that of histology, with measurements of the retinal nerve fiber layer to detect nerve swelling and predict the visual outcome^18^. The strengths of our study are that the sample size, although small, includes an acceptable number of patients in a single tertiary center, and the long-term follow-up, as it allows the evaluation of the natural history of the disease.

In conclusion, the diagnosis of CRION is challenging and should be kept in mind. Prompt diagnosis, adequate treatment, and close follow-up are essential to prevent disabling sequelae in these patients.

## References

[1] Raimundo M, Fonseca C, Lemos J, Fonseca P (2018). Central serous chorioretinopathy as a cause of vision loss in chronic relapsing inflammatory optic neuropathy. Am J Ophthalmol Case Rep.

[2] Kidd D, Burton B, Plant GT, Graham EM (2003). Chronic relapsing inflammatory optic neuropathy (CRION). Brain.

[3] Petzold A, Plant GT (2014). Chronic relapsing inflammatory optic neuropathy: A systematic review of 122 cases reported. J Neurol.

[4] Saini M, Khurana D (2010). Chronic relapsing inflammatory optic neuropathy. Ann Indian Acad Neurol.

[5] Koraszewska-Matuszewwska B, Samochowiec-Donocik E, Rynkiewicz E (1995). Optic Neuritis in children and adolescents. Klin Oczna.

[6] Abdullah S Wong WF, Tan CT (2017). The prevalence of anti-aquaporin 4 antibody in patients with idiopathic inflammatory demyelinating diseases presented to a tertiary hospital in malaysia: presentation and prognosis. Mult Scler Int.

[7] Petzold A, Pittock S, Lennon V, Maggiore C, Weinshenker BG, Plant GT (2010). Neuromyelitis optica-IgG (aquaporin-4) autoantibodies in immune mediated optic neuritis. J Neurol Neurosurg Psychiatry.

[8] Jarius S, Frederikson J, Waters P, Paul F, Akman-Demir G, Marignier R (2010). Frequency and prognostic impact of antibodies to aquaporin-4 in patients with optic neuritis. J Neurol Sci.

[9] Liu H, Zhou H, Wang J, Xu Q, Wei S (2019). Antibodies to myelin oligodendrocyte glycoprotein in chronic relapsing inflammatory optic neuropathy. Br J Ophthalmol.

[10] Petzold A, Woodhall M, Khaleeli Z, Tobin WO, Pittock SJ, Weinshenker BG (2019). Aquaporin-4 and myelin oligodendrocyte glycoprotein antibodies in immune-mediated optic neuritis at long-term follow-up. J Neurol Neurosurg Psychiatry.

[11] Stiebel-Kalish H, Hammel N, Van Everdingen J, Huna-Baron R, Lee AG (2010). Intravenous immunoglobulin in recurrent-relapsing inflammatory optic neuropathy. Can J Ophthalmol.

[12] Papp V, Langkilde AR, Blinkenberg M, Schreiber K, Jensen PEH, Sellebjerg F (2018). Clinical utility of anti-MOG antibody testing in a Danish cohort. Mult Scler Relat Disord.

[13] Sahraian MA, Moghadasi AN, Azimi AR, Asgari N, Akhoundi FH, Abolfazli R (2017). Diagnosis and management of Neuromyelitis Optica Spectrum Disorder (NMOSD) in Iran: A consensus guideline and recommendations. Mult Scler Relat Disord.

[14] Vern VCN, Thavaratnam LK (2018). Rituximab in chronic relapsing inflammatory optic neuropathy (CRION). Med J Malaysia.

[15] Arzani M, Sahraian MA, Rezaei H, Moghadasi AN (2017). Recurrent isolated optic neuritis: A study on 22 patients. Iran J Neurol.

[16] Hervás-García JV, Pagani-Cassara F (2019). Chronic relapsing inflammatory optic neuropathy: A literature review. Rev Neurol.

[17] Lee HJ, Kim B, Waters P, Woodhall M, Irani S, Ahn S (2018). Chronic relapsing inflammatory optic neuropathy (CRION): a manifestation of myelin oligodendrocyte glycoprotein antibodies. J Neuroinflammation.

[18] Iorga RE, Moraru A, Ozturk MR, Costin D (2018). The role of Optical Coherence Tomography in optic neuropathies. Rom J Ophthalmol.

